# Neuromodulation Strategies to Reduce Inflammation and Improve Lung Complications in COVID-19 Patients

**DOI:** 10.3389/fneur.2022.897124

**Published:** 2022-07-14

**Authors:** Christopher J. Czura, Marom Bikson, Leigh Charvet, Jiande D. Z. Chen, Manfred Franke, Marat Fudim, Eric Grigsby, Sam Hamner, Jared M. Huston, Navid Khodaparast, Elliot Krames, Bruce J. Simon, Peter Staats, Kristl Vonck

**Affiliations:** ^1^Convergent Medical Technologies, Inc., Oyster Bay, NY, United States; ^2^Department of Biomedical Engineering, The City College of New York, New York, NY, United States; ^3^Department of Neurology, NYU Grossman School of Medicine, New York, NY, United States; ^4^Division of Gastroenterology and Hepatology, University of Michigan School of Medicine, Ann Arbor, MI, United States; ^5^NeuronOff, Inc., Cleveland, OH, United States; ^6^Division of Cardiology, Duke Clinical Research Institute, Duke University, Durham, NC, United States; ^7^Neurovations, Napa, CA, United States; ^8^Cala Health, Burlingame, CA, United States; ^9^Departments of Surgery and Science Education, Zucker School of Medicine at Hofstra/Northwell, Institute of Bioelectronic Medicine, Feinstein Institutes for Medical Research, Manhasset, NY, United States; ^10^Spark Biomedical, Inc., Dallas, TX, United States; ^11^Pacific Pain Treatment Center, Napa, CA, United States; ^12^ElectroCore, Inc., Basking Ridge, NJ, United States; ^13^National Spine and Pain, ElectroCore, Inc., Jacksonville, FL, United States; ^14^Department of Neurology, Ghent University Hospital, Ghent, Belgium

**Keywords:** vagus nerve (VN) stimulation, sacral nerve electrical stimulation, COVID-19, cytokine storm, acute respiratory distress (ARDS), cranial nerve stimulation, non-invasive

## Abstract

Since the outbreak of the COVID-19 pandemic, races across academia and industry have been initiated to identify and develop disease modifying or preventative therapeutic strategies has been initiated. The primary focus has been on pharmacological treatment of the immune and respiratory system and the development of a vaccine. The hyperinflammatory state (“cytokine storm”) observed in many cases of COVID-19 indicates a prognostically negative disease progression that may lead to respiratory distress, multiple organ failure, shock, and death. Many critically ill patients continue to be at risk for significant, long-lasting morbidity or mortality. The human immune and respiratory systems are heavily regulated by the central nervous system, and intervention in the signaling of these neural pathways may permit targeted therapeutic control of excessive inflammation and pulmonary bronchoconstriction. Several technologies, both invasive and non-invasive, are available and approved for clinical use, but have not been extensively studied in treatment of the cytokine storm in COVID-19 patients. This manuscript provides an overview of the role of the nervous system in inflammation and respiration, the current understanding of neuromodulatory techniques from preclinical and clinical studies and provides a rationale for testing non-invasive neuromodulation to modulate acute systemic inflammation and respiratory dysfunction caused by SARS-CoV-2 and potentially other pathogens. The authors of this manuscript have co-founded the International Consortium on Neuromodulation for COVID-19 to advocate for and support studies of these technologies in the current coronavirus pandemic.

## Introduction

Preventative strategies to reduce infections of the coronavirus, SARS-CoV-2, including wearing masks (N95 most effective) and face shields, avoiding prolonged exposure to infected individuals, avoiding confined indoor spaces, quarantines, and vaccines and boosters, have proven successful. While disease-modifying therapeutic strategies for coronavirus-induced disease-2019 (COVID-19), including the antiviral remdesivir (1), are emerging, the contagiousness of the virus and the morbidity and mortality associated with COVID-19 continue to place an urgent demand on the development of new therapies. Presently worldwide, nearly 450 million persons have been infected worldwide, 6 million of whom have succumbed to the disease (https://coronavirus.jhu.edu/map.html). Severe cases of COIVD-19 are often associated with a “cytokine storm,” which may cause dysfunction of the lungs and other organs. Studies investigating pharmacological therapies targeting the immune and respiratory systems are ongoing.

The human immune and respiratory systems are regulated by the central nervous system (CNS), and therapeutic targeting of these neural pathways is potentially one approach to regulate important aspects of the pathologic sequelae of COVID-19. Neuromodulation directly targets the nervous system through the application of electric or magnetic fields to neuronal tissue, including peripheral nerve fibers and directly to the brain. Neuromodulation may therefore provide alternative therapeutic strategies for the neurobiological systems involved in the pathophysiology of SARS-CoV-2 infections.

This review provides an overview of the role of the nervous system in immune and respiratory control, current understanding of how neuromodulatory techniques may be effective in immune or pulmonary disorders, and provides a future perspective of its role in the treatment of COVID-19. The authors of this review formed the International Consortium on Neuromodulation for COVID-19 (www.covidneuromod.org) in April, 2020, in response to the pandemic with the understanding that neuromodulation, particularly vagus nerve stimulation (VNS) or sacral nerve modulation (SNM), may be able to block infection-induced inflammation through the cholinergic antiinflammatory pathway (CAP). Now including 30 members on three continents, this group was formed to advocate for and support the scientific study of neuromodulation technologies for treatment of excessive inflammation and pulmonary dysfunction in this and future pandemics when appropriate. The authors provide recommendations on clinical trial endpoints, as well as recommended publication guidelines in an effort to standardize technology characterizations.

## The Hyperinflammatory State in COVID-19

In up to 30% of mortality caused by SARS-CoV-2, a severe systemic inflammatory response known as a “cytokine storm” is implicated in multi-system organ failure (2, 3). These cases are characterized by high concentrations of circulating proinflammatory cytokines, including TNF, IL-6, IL-7; the inflammatory chemokines CCL2, CCL3 and CXCL10 (4); and acute-phase proteins including C-reactive protein and ferritin are also associated with disease severity (4, 5). Clinically, the most typical phenotype of severe COVID-19 is acute respiratory distress syndrome (ARDS), which affects 3.4% of infected patients overall and 15.6–17.0% of severely infected patients (6). The COVID-19 hyperinflammatory response may induce disseminated intravascular coagulation through both endothelial cell activation and impairment of endogenous anticoagulant pathways (7–12). This disease pathophysiology may pose specific problems for individuals with preexisting cardiopulmonary conditions, including hypertension and chronic obstructive pulmonary disease.

## Role of the Nervous System in Immune and Respiratory Control

The mammalian nervous and immune systems evolved to maintain homeostasis and protect the host against tissue injury and infection (13). Autonomic nerves monitor infection and inflammation and form homeostatic reflexes to regulate host responses (14, 15). An important neural conduit for monitoring and regulating immune responses is the vagus nerve (VN), the body's longest cranial nerve. Peripheral vagal fibers sense local cytokines and damage- and pathogen-associated molecular patterns (DAMPs, PAMPs), which activate neural regulatory reflexes (16, 17). The brain can respond to active inflammation via glucocorticoid release from the hypothalamic-pituitary-adrenal (HPA) axis, or through activation of the CAP (18). The CAP comprises efferent VN fibers innervating the spleen via the abdominal celiac ganglion and splenic nerve. The role of sympathetic nerves in regulating inflammation and pulmonary function are reviewed elsewhere (15, 19–21).

Electrical VNS was first shown to protect against excessive inflammation in 2000, when Borovikova et al. reported that VNS reduces systemic proinflammatory cytokine production and maintains blood pressure in a rodent model of endotoxic shock (22). Electrically or pharmacologically stimulating the CAP protects against excessive systemic inflammation, maintains tissue perfusion, and preserves vital organ function in models of arthritis, burns, colitis, hemorrhagic shock, polymicrobial intraabdominal sepsis, and stroke (23–34). Importantly, disruption of CAP signaling delays resolution of inflammation and bacterial clearance in experimental peritonitis (35). Specific CAP components also play key roles in regulation of circulation, microvascular contractility, and viral infection clearance in experimental models (36, 37).

Molecular and functional mapping of the CAP have revealed that VNS inhibits release of pro-inflammatory mediators via acetylcholine-releasing T lymphocytes expressing choline acetyltransferase (ChAT^+^) (38). Local acetylcholine secretion in spleen inactivates cytokine-producing macrophages through surface α7 nicotinic acetylcholine receptor subunits (37). Pro-inflammatory cytokine production in spleen contributes significantly to overall systemic inflammation (39). Elimination of these cellular or molecular components via genetic deletion or surgical disruption abolishes CAP activity and results in significantly elevated serum cytokine levels (40).

In addition to providing neural regulation of inflammation, the VN also provides the dominant autonomic innervation of the airways, carrying fibers that are responsible for both bronchoconstriction and relaxation through distinct sets of pulmonary nerve branches (41–43). Low-intensity VNS relaxes airway smooth muscle in animal models of bronchoconstriction by activating the HPA axis to release catecholamines from the adrenal cortex (41, 44, 45). These in turn bind to β-receptors on bronchial smooth muscle causing relaxation. Measured concentrations of epinephrine and norepinephrine were sufficient to relax smooth muscle, but insufficient to cause significant cardiac effects (46, 47).

The sacral nerve (SN) is part of the parasympathetic nervous system that also holds therapeutic potential for pathological inflammatory processes. Both afferent and efferent SN fibers innervate the pelvic organs including colon, rectum, urinary bladder and genital organs. Sacral nerve modulation (SNM) has been used to treat diseases of the pelvic organs, including pain, urinary retention, overactive bladder and fecal incontinence (48, 49). The mechanisms of action of SNM are not completely understood. Limited findings suggest that SNM ameliorates overactive bladder by blocking C fiber activities or overactivity (50). In treating urinary retention, SNM seems to stimulate relaxation of pelvic floor muscles and the urethra, facilitating micturition (51, 52). The mechanism involved in SNM-induced improvement in fecal incontinence is also poorly understood, with most research findings suggesting that SNM stimulates afferent fibers from the rectum, pelvic floor and anal sphincter, resulting in reduced activation of C fibers during rectal filling and inhibiting inputs from the rectum to the pontine center (53). SNM in patients with fecal incontinence may activate somatic afferent fibers to inhibit colonic motility and enhance internal anal sphincter pressure (54).

Recently, SNM has been shown to reduce intestinal inflammation in rodent models of inflammatory bowel diseases, suppress a number of proinflammatory cytokines including TNF, and enhance anti-inflammatory cytokines such as IL-10 in both intestinal tissues and in circulation (55, 56). The anti-inflammatory effect of SNM on the colon is mediated via the spinal afferent and vagal efferent pathways evidenced by the following factors (57): (1) SNM activates neurons in the nucleus tractus solitaries (57, 58); (2) SNM increases vagal efferent activity as assessed by the spectral analysis of heart rate variability and plasma level of pancreatic polypeptide (55); (3) bilateral subdiaphragmatic vagotomy almost completely abolishes the anti-inflammatory effect of SNM on the colon (57); and (4) SNM induces the release of acetylcholine in the colon (55). These findings suggest that SNM shares a similar vagal efferent pathway as VNS. Indeed, a comparative study has shown that the anti-inflammatory effect of SNM was similar to that of VNS and the combination of SNM and VNS did not show a synergistic effect in comparison with SNM or VNS alone (59, 60).

Together, these findings suggest that VNS or SNM enhance vagal efferent activity and have systemic anti-inflammatory effects. Several VNS technologies are being investigated, some for use in COVID-19, as summarized below.

## Vagus Nerve Modulation in Clinical Practice and Research

Electrical signaling between the CNS and individual organ systems is essential to organ function. Homeostasis is continually monitored and regulated through a bi-directional feedback loop that senses the physiological status of organ function, central nervous system processing of the sensory information, and appropriate feed-back to the organs. Therapeutic neuromodulation aims to restore a diseased organ system by interfering with or augmenting its neural control mechanisms via targeted application of external energy sources, typically electrical or magnetic. Neuromodulation technologies have been developed to treat neuropsychiatric disorders and conditions where the nervous system is known to play an important role, including epilepsy, depression and pain (61, 62). Of note, VNS utilizing a surgically implanted electrical pulse generator is an established clinical therapy for the treatment of medically refractory epilepsy and depression. Since 1988, more than 100,000 patients have received this FDA-approved and CE-marked therapy, and long-term follow-up studies demonstrate overall safety of the approach (63).

Functional mapping of the CAP has provided a foundation for clinical trials to evaluate the efficacy of VNS to treat inflammatory diseases. Several studies, albeit with a limited number of patients included, show encouraging data on use of electrical VNS for the treatment of chronic inflammation, e.g., rheumatoid arthritis and Crohn's disease (64–66). VNS significantly reduced whole blood TNF, IL-6, and IL-1β concentrations, and decreased scores on a validated disease activity composite score (DAS28-CRP score) (65). Likewise, non-invasive cervical VNS was tested in two clinical trials for treating acute asthma patients presenting to the emergency department who were unresponsive to 1 h of standard of care therapy (67–69). In both studies, VNS significantly improved two measures of airway patency, FEV_1_ and perceived work of breathing, after 15, 30 and 60 min (compared with a matched control group), with no reported serious adverse events. These data suggest a possible, novel treatment for COVID-19 patients suffering from acute respiratory dysfunction. However, care must be exercised in choosing stimulation parameters because high intensity cervical VNS, such as is used to treat epilepsy and depression, is capable of inducing bronchoconstriction, which is reversed upon cessation of stimulation (70).

## Neuromodulation Technologies for COVID-19

In light of the available mechanistic experimental data on the capacity of neuromodulation techniques to attenuate excessive acute and chronic inflammation, and induce bronchodilation; the clinical safety record of electrical VNS; and the encouraging reports on reduced inflammation with chronic implantation of an electrical vagus nerve stimulator in clinical trials, it is reasonable to consider whether neuromodulation devices may be useful tools to reduce the morbidity and mortality of COVID-19 patients (71–79). A significant challenge for applications in acute-onset excessive inflammation is the delivery method of VNS, as it is currently impractical to implant highly symptomatic COVID-19 patients with an active device that is only needed for acute treatment. However, less invasive neuromodulation techniques, either percutaneous or transcutaneous, may be utilized (Tables 1–4). These methods may be technically suitable for acute use, but their clinical feasibility and efficacy in COVID-19 patients are just beginning to be evaluated. Results from two recent COVID-19 studies have been published, results are pending from several ongoing clinical trials, and a number have been discontinued (discussed below).

**Table 1:** Examples of commercially available devices that are currently being tested or have recently been tested in COVID-19 trials.

**Device**	**Manufacturer/ Distributor**	**Neural target**	**Neural interface**	**Regulatory status**	**Indication/Use**	**URL**	**ClinicalTrials.gov Identifiers**
gammaCore	electroCore, Inc.	CVN	Transcutaneous, unilateral	FDA cleared, CE marked	Cluster headache (FDA & CE), migraine (FDA & CE), asthma (CE), airway reactivity (CE)	gammacore.com	COVID-19: NCT04368156, NCT04382391 Inflammation-related: NCT05315739, NCT04143269, NCT05165108, NCT01627301, NCT02992899, NCT04935697, NCT04556552, NCT04099992 Pulmonary-related: NCT03869008, NCT04935697, NCT01679314, NCT00762931 (Note A) Other: Note B
AuriStim	Multisana	ABVN	Percutaneous, unilateral	CE marked	Wellness	multisana.at	COVID-19: NCT05058742 Inflammation-related: none Pulmonary-related: none Other: NCT05131334
Vitality Smartcable	Nemechek Technologies, Inc.	ABVN	Transcutaneous, unilaterlal	None	Wellness	nemechektechnologies.shop	COVID-19: NCT04379037 Inflammation-related: none Pulmonary-related: none Other: none

*Note A: some pulmonary studies were conducted on alphaCore, a predecessor device to gammaCore that had a similar stimulation waveform. Note B: gammaCore trials listed here are a subset of 34 total trials registered in ClinicalTrials.gov; the remainder are unrelated to pathogenic mechanisms of acute COVID-19 and are not listed here due to space constraints. ABVN, auricular branch of the vagus nerve; CVN, cervical vagus nerve*.

**Table 2:** Examples of commercially available devices that are currently being tested in non-COVID-19 trials, but are assessing endpoints related to pathogenic mechanisms of COVID-19.

**Device**	**Manufacturer/ Distributor**	**Neural target**	**Neural interface**	**Regulatory status**	**Indication/Use**	**URL**	**ClinicalTrials.gov Identifiers**
NEMOS	T-VNS technologies	ABVN	Transcutaneous, unilateral	CE marked	Epilepsy	nemos.t-vns.com	COVID-19: none Inflammation-related: NCT01924780, NCT02359188 Pulmonary-related: none Other: NCT02409069, NCT04632134, NCT03722901, NCT02620176, NCT02177890, NCT02156817, NCT05180916, NCT03615209, NCT05157334
ParaSym	Parasym	ABVN	Transcutaneous, unilateral	CE marked, FDA IDEs issued	N/A	parasym.co	COVID-19: Inflammation-related: NCT03392649, NCT02548754, NCT05350150 Pulmonary-related: Other: NCT03930914, NCT05034419, NCT04682704, NCT05341544, NCT05350150 Post-COVID/PASC: NCT05225220
TENStem w/ auricular electrodes	Schwa-Medico	Auricular	Transcutaneous	CE Marked	Wellness	TENS unit: https://www.schwa-medico.com/en/electrostimulation/tens/tenstem-eco-basic/schwa-medico-electrostimulation-tens-tenstem-eco-basic.html TENS accessories: schwa-medico.com/en/electrostimulation/tens-ems-accessories/	COVID-19: none Inflammation-related: NCT04520516, NCT04286373 Pulmonary-related: none Other: none

*ABVN, auricular branch of the vagus nerve; ATN, auriculotemporal nerve; CN, cranial nerves; IDEs, investigational device exemptions; GAN, greater auricular nerve; tDCS, transcranial direct current stimulation; tES, transcranial electrical stimulation; EEG, electroencephalography; FUS, focused ultrasound*.

**Table 3:** Examples of commercially available devices that are currently being tested in trials unrelated to COVID-19, but which have neural mechanisms of action that could be related to the pathological sequelae of COVID-19.

**Device**	**Manufacturer/ Distributor**	**Neural target**	**Neural interface**	**Regulatory status**	**Indication/Use**	**URL**	**ClinicalTrials.gov Identifiers**
Alpha-Stim	Alpha-Stim	GAN	Transcutaneous, unilateral	FDA cleared, CE marked	Anxiety, insomnia, depression, pain	alpha-stim.com	COVID-19: Inflammation-related: Pulmonary-related: Other: NCT04963907, NCT03757494, NCT04770181, NCT03060122, NCT02901080, NCT01533415, NCT04115033, NCT03210155, NCT00723008, NCT04587531
Cefaly	Cefaly	Trigeminal	Transcutaneous	FDA cleared, CE marked	Migraine (acute treatement and prevention)	cefaly.com	COVID-19: none Inflammation-related: none Pulmonary-related: none Other: NCT02546362, NCT04838067, NCT03217968, NCT02342743, NCT02411513, NCT02616978, NCT03125525, NCT02590939, NCT03465904, NCT05200897, NCT02125422, NCT02122237, NCT02307071, NCT02122757, NCT02462395
electro-detox	Soterix Medical, Inc	tDCS	Percutaneous, unilateral	FDA cleared	Opioid withdrawal syndrome	soterixmedical.com	COVID-19: Inflammation-related: Pulmonary-related: Other:
Fisher Wallace Stimulator	Fisher Wallace	tDCS	Transcutaneous	FDA cleared	Depression, anxiety, insomnia	fisherwallace.com	COVID-19: none Inflammation-related: none Pulmonary-related: none Other: NCT04751864, NCT04627480, NCT04541563, NCT02163967, NCT01325532
Flow	Flow Neuroscience	tDCS	Transcutaneous	CE Marked	Depression	flowneuroscience.com	COVID-19: none Inflammation-related: none Pulmonary-related: none Other: NCT05202119
Halo	Halo	tDCS	Transcutaneous	N/A	Neuroplasticity	haloneuro.com	COVID-19: none Inflammation-related: none Pulmonary-related: none Other: NCT04883229
NSS-2 BRIDGE	Innovative Health Solutions	CN V, VII, IX, and X	Percutaneous, unilateral	FDA cleared	Opioid withdrawal syndrome	i-h-s.com/ products/ bridge/	COVID-19: none Inflammation-related: none Pulmonary-related: none Other: NCT03555266, NCT03834142, NCT03830307, NCT04365465, NCT03931330, NCT04162145, NCT04325659, NCT03762798
Xen	Neuvana	ABVN	Transcutaneous, bilateral	CE marked	Wellness	neuvanalife.com	COVID-19: none Inflammation-related: none Pulmonary-related: none Other: NCT05132881
PainX or tCDS-LTE	Soterix Medical, Inc	tDCS	Transcranial, bilateral	CE marked	Fibromyalgia, migraine	soterixmedical.com	COVID-19: none Inflammation-related: none Pulmonary-related: none Other: NCT04994821, NCT04781127, NCT03833583, NCT02648542, NCT02540109, NCT01651884
Starstim	Neuroelectrics	tES plus EEG	Transcutaneous	CE Marked	Research tool	neuroelectrics.com	COVID-19: none Inflammation-related: none Pulmonary-related: none Other: NCT05205915, NCT04770337, NCT02866240, NCT03943979, NCT03144102
Sparrow	Spark Biomedical, Inc.	ABVN and ATN	Transcutaneous, unilateral	FDA cleared	Opioid withdrawal syndrome	sparkbiomedical.com	COVID-19: none Inflammation-related: none Pulmonary-related: none Other: NCT04731935, NCT05129020, NCT05053503, NCT04588519, NCT04075214

*ABVN, auricular branch of the vagus nerve; ATN, auriculotemporal nerve; CN, cranial nerves; GAN, greater auricular nerve; tDCS, transcranial direct current stimulation; tES, transcranial electrical stimulation; EEG, electroencephalography*.

**Table 4:** Examples of devices with neural mechanisms that may be related to modulating the pathological sequelae of COVID-19, but which have not yet been assessed for relevant endpoints.

**Device**	**Manufacturer/ Distributor**	**Neural target**	**Neural interface**	**Regulatory status**	**Indication/Use**	**URL**	**Clinicaltrials.gov Identifiers**
Acuson Sequoia 512	Seimens	FUS	Transdermal	FDA cleared for imaging	Imaging	avantehs.com/p/siemens-acuson-sequoia-512-lcd-ultrasound-machine/1083	COVID-19: none Inflammation-related: none Pulmonary-related: none Other: imaging only
AGISTIM and ASP needles	Sedatelec	ABVN or ATN	Percutaneous, unilateral or bilateral	CE marked	N/A	sedatelec.com	COVID-19: none Inflammation-related: none Pulmonary-related: none Other: none
Dolphin	Dolphin Neurostim	ABVN	Transcutaneous/ percutaneous, unilateral	Health Canada expanded use authorization	Wellness	dolphinmps.com	COVID-19: none Inflammation-related: none Pulmonary-related: none Other: none
LOGIQ E9 with C1-6 transducer	GE Healthcare	FUS	Transdermal	FDA cleared for imaging	Imaging	gehealthcare.com/products/ultrasound/logiq/logiq-e9-with-xdclear	COVID-19: none Inflammation-related: none Pulmonary-related: none Other: imaging only
VaguStim	VaguStim	ABVN	Transcutaneous, unilateral or bilateral	Unknown	N/A	vagustim.io	COVID-19: none Inflammation-related: none Pulmonary-related: none Other: NCT04520516, NCT05088135, NCT05370027

*ABVN, auricular branch of the vagus nerve; FUS, focused ultrasound*.

### Transcutaneous Cervical VNS

gammaCore (electroCore, Inc., Rockaway, NJ USA) is a handheld, transcutaneous cervical vagus nerve stimulator that has been FDA approved and CE marked for cluster headache and migraine. Functional magnetic resonance imaging has shown that gammaCore specifically modulates various brain structures, including the locus coeruleus (LC) and the NTS, and that these activation patters are similar between invasive VNS, non-invasive cervical VNS, and transcutaneous auricular VNS (described further below) (80–82). In off-label studies, gammaCore reduced expression of interleukin [IL]-1β, IL-6, IL-8, tumor necrosis factor [TNF]), macrophage inflammatory protein [MIP]-1α, and monocyte chemoattractant protein [MCP]-1, and increased cardiac vagal tone (83–88). gammaCore also improved lung function in treatment-refractory emergent asthma (69). Together, these observations suggest that gammaCore may have therapeutic utility in COVID-19. Indeed, the FDA issued an Emergency Use Authorization for asthma patients experiencing exacerbation of symptoms when infected with the novel coronavirus (89).

### Transcutaneous Auricular VNS

Recently, several studies examined the function of the auricular branch of the vagus nerve (ABVN) as a potential non-invasive method to activate the NTS and associated structures. Functional magnetic resonance imaging has shown specific modulation of various brain structures following transcutaneous auricular neurostimulation (tAN), including the NTS and nucleus spinalis of the trigeminal nerve (90, 91). Thus, the ABVN serves as a non-invasive access point to the CNS to deliver information, as similar to cervical VNS. Stimulation of the ABVN downregulated expression of IL-6, IL-1β, IP-10, MIP-1α, TNF, and C-reactive protein (CRP); and increased expression of the anti-inflammatory cytokine IL-10 (92–94). These stimulation paradigms also reduced incidence of pneumonia and hospital length of stay following lung lobectomy; reduced atrial fibrillation burden; and improved disease activity scores (DAS28-CRP) in rheumatoid arthritis (92–94). Electrical stimulation parameters that have typically shown results have focused on the left ear, using stimulation pulses of ~200 ms, delivered at 20 Hz, with current at or somewhat below a level that causes discomfort; and treatment paradigms have delivered as little as 1 h of stimulation daily for 6 months or less (93). In March of 2021, Health Canada issued an expanded use authorization to Dolphin Neurostim for the acute treatment of adult patients with known or suspected COVID-19 who are experiencing exacerbation of asthma-related dyspnea and reduced airflow (95).

### Electrical and Magnetic Transcranial Approaches

In addition to modulation of neural pathways that directly modify end-organ function, top-down neuromodulation technologies target central neural structures to achieve similar effects. Low-intensity transcranial electrical stimulation (tES) approaches include transcranial Direct Current Stimulation (tDCS) (96) and transcranial Alternating Current Stimulation (tACS) (97). Transcranial Magnetic Stimulation (TMS) applies varied pulse waveforms (98). Transcranial approaches share the goal of directly activating intra-cranial brain structures (99), though ancillary peripheral [e.g., cranial nerve (100)] stimulation cannot be excluded. The rational for tES, and especially tDCS, for COVID-19 brain disorders have been reviewed (77, 101, 102). Several early trials and case series suggest efficacy of tDCS of post-acute sequelae SARS-CoV-2 infection (PASC)/Long-COVID (103–105).

Noninvasive brain stimulation has the broad potential to manage some of the complications of COVID-19 by stimulating the regions involved in the regulation of systemic anti-inflammatory responses and recovery of respiration. There is a bidirectional influence between the brain and immune response (106). Deep brainstem and forebrain regions mediate the immune response throughout the body and can be potential targets for noninvasive neuromodulatory approaches such as tES/TMS. While it is impractical to activate deep regions selectivity (e.g., without activating superficial cortex) by tES or TMS, deep brain regions can certainly be reached by tES (107–110) current and through connectivity to cortical activated areas (111, 112). Cortical regions are conventionally targeted with tES/TMS, including frontal (113) and temporal regions (114) that can be used to influence the systemic immune response and prevent neuroinflammation (106). Furthermore, tES/TMS may be investigated for application in the restoration of respiratory function in recovery (115). There are thus multiple pathways by which tES/TMS can address immediate and long-term COVID-19 morbidity, but subject to direct experimental testing in COVID-19 patients these links remain indirect.

### Ultrasound-Guided Percutaneous Electrodes

One means of direct, percutaneous stimulation was recently demonstrated in mice, using low intensity “imaging” ultrasound to guide a percutaneous needle electrode within proximity to the cervical vagus nerve, but can be extrapolated to other targets as well. Using relatively low energy electrical stimulation, biomarkers of vagus engagement, including bradycardia and brainstem c-FOS activation was confirmed. Percutaneous VNS modulated endotoxin-induced TNF production, CNS inflammation, as well as cognitive dysfunction (116). Coupled with the established long-duration of anti-inflammatory effects of VNS after a single treatment (117, 118), this data demonstrates the feasibility of using ultrasound-guided percutaneous electrical stimulation to modify cytokine storm. Translation of this technique to humans should be trivial. While minimally invasive, this procedure would require a trained health care provider to perform and would be limited to in-clinic use. This technique was used to stimulate the vagus nerve in the Miner clinical study of VNS on bronchoconstriction patients (67).

### Focused Ultrasound

A promising non-invasive technology is focused ultrasound, which uses acoustic pressure waves to transmit mechanical and heat energy to neural tissues, rather than electrical energy. The long history of focused ultrasound to therapeutically modulate neural tissue since the 1950's, in both central and peripheral systems, has been reviewed elsewhere (119). These established techniques can be used for destructive targeted ablation of tissues, to activate neurons, or to suppress the activation of neural circuits. The elucidation of neural circuits that control inflammation have generated a number of intervention points that may be targeted by focused ultrasound, including deep brain structures, cranial nerves, peripheral nerves, and end organs.

The spleen and its attendant nerves are a major interaction nexus between the neural and immune systems (38, 39, 120), and has recently been explored as a target for focused ultrasound intervention in rodents by several groups (121–124). Focused ultrasound targeting the spleen reduced endotoxin-induced TNF (123), retention of renal form and function following ischemia reperfusion injury, including reduction in renal accumulation of neutrophils and dendritic cells (121, 122), reduction in plasma creatinine following cecal ligation and puncture (122), and attenuation of disease manifestation in a serum transfer model of inflammatory arthritis (124). Demonstrating the potential to tailor a specific outcome by modulating a specifically targeted tissue, focused ultrasound on glucose sensory neurons in the liver prevented hyperglycemia following endotoxin exposure (123). While extremely promising as a non-invasive technique to modulate inflammation and its sequelae, these studies have demonstrated that both the dose delivered and the sub-organ location targeted have a large impact on the physiological outcome, relegating its use to a trained health care provider in-clinic. Focused ultrasound as a modulator of inflammation still requires demonstration of translation to humans.

## Alternative Mechanisms

The exact mechanisms of VNS are not fully understood. In addition to activating efferent CAP fibers to the spleen, VNS is believed to proportionally activate afferent fibers (40, 125). Vagal afferents carry sensory information to the dorsal medullary complex, in particular to the nucleus of the solitary tract (NTS). Activation of the NTS plays an important role in modulating cardiopulmonary function (126–128). Therapeutic mechanisms in epilepsy and depression may involve regulating the serotonergic and/or noradrenergic projections from the raphe and LC, respectively, which in turn modulates activity in the hypothalamus, thalamus, insular cortex, and cerebellum, hippocampus, amygdala, and posterior cingulate gyrus (129, 130). These structures are associated with the regulation of mood and emotion, seizure activity, anxiety, intestinal activity, satiety, and pain perception (131–134). Other potential, centrally mediated mechanisms for VNS include elevation of gamma aminobutyric acid (GABA) levels in the brain stem (135), desynchronization of cortical and thalamocortical network activity (125), suppression of cortical spreading depression (136), altering expression of brain-derived neurotrophic factor and other growth factors and increasing norepinephrine in prefrontal cortex (137), and altering brain waste clearance dynamics (138).

## Recent COVID-19 Trials

There are currently six studies listed on ClinicalTrials.gov assessing the effects of VNS on immune and/or pulmonary function in COVID-19 (Table 5). Two of the trials used gammaCore, and thus targeted the cervical vagus nerve transcutaneously. Four trials employed several different auricular vagus nerve stimulation devices. Two studies are completed, three are open to enrollment or active but not recruiting, and one terminated early due to low recruitment rates.

**Table 5:** Clinical trials of vagus nerve stimulation in COVID-19 that are registered at ClinicalTrials.gov and that include proposed mechanisms of action related to immune and/or pulmonary function.

**Title**	**Conditions**	**Neural target**	**Device**	**NCT number**	**Status**
Study Assessing Vagus Nerve Stimulation in CoViD-19 Respiratory Symptoms (SAVIOR-1)	Covid-19	Cervical vagus	gammaCore	NCT04368156	Completed
Study Assessing Vagus Nerve Stimulation in CoViD-19 Respiratory Symptoms (SAVIOR-2)	COVID|Corona virus Infection|Respiratory Failure|Respiratory Distress Syndrome, Adult|ARDS, Human|SARS (Severe Acute Respiratory Syndrome)	Cervical Vagus	gammaCore Sapphire	NCT04382391	Active, not recruiting
Non-invasive Nervus Vagus Stimulation in Patients With COVID-19 and ARDS	COVID-19|ARDS|Cytokine Storm	Auricular vagus	AuriStim	NCT05058742	Recruiting
Neuromodulation With Percutaneous Electrical Nerve Field Stimulation for Adults With COVID-19	COVID-19	Auricular vagus	NSS-2 BRIDGE	NCT04514627	Recruiting
Impact of Auricular vagus Nerve Neuromodulation on COVID-19 Positive Inpatients Outcome	Covid19|SARS-CoV Infection	Auricular vagus	Procedure: Auricular neuromodulation (acupuncture)	NCT04341415	Terminated
Vagus Nerve Stimulation ARDS Prevention Trial for COVID-19 Hospitalized Patients	Severe Acute Respiratory Syndrome Coronavirus 2	Auricular Vagus	Nemechek Vitality Smartcable	NCT04379037	Completed

*No trials are currently registered exploring other neuromodulation strategies for these mechanisms of action in COVID-19*.

The use of cervical non-invasive VNS in COVID-19 was first published in a case report of two COVID-19-positive subjects, which provided preliminary observations that transcutaneous cervical VNS reduced the use of opioid and cough suppressant medications and relieved symptoms of chest tightness and shortness of breath (139). Two randomized control trials of gammaCore in inpatients with COVID-19 and respiratory distress have since been initiated: SAVIOR-1, performed in Valencia, Spain, was a prospective, randomized controlled trial (RCT) of VNS vs. standard of care, which evaluated a total of 90 patients, 45 in each arm. Improvement in respiratory function and proinflammatory cytokine levels were measured following VNS (NCT04368156) (140). SAVIOR-2, performed at Allegheny Health Network, was scheduled to study 60 patients and also followed respiratory parameters and cytokines (NCT04382391). This study closed to enrollment after 21 subjects due to low case burdens resulting in low recruitment rates.

The remaining studies employed devices targeting the auricular branch of the vagus nerve either in the cymba concha or the tragus. The results of the first study suggest that auricular VNS results in infrequent mechanical ventilation and a high rate of survival, though it should be noted that this trial in 51 subjects was a single-arm, uncontrolled, open-label, observational trial (141). Studies of two additional auricular VNS devices are actively recruiting, and results are not yet available. A French study is assessing auricular nerve stimulation via four percutaneously placed leads on each tragus and is being compared to sham (no needle placement; NCT04341415). Enrollment has been halted due to a low case load (142).

## Future Perspectives

COVID-19 is a deadly disease, the most serious effects of which appear to be hyperactivation of an acute inflammatory response (“cytokine storm”) and compromised lung function. To date, pharmacological strategies have not been generally successful in modulating the cytokine response and clinical sequellae to SARS-CoV-2, with the exception of dexamethasone, a non-specific anti-inflammatory corticosteroid that reduces mortality by one-third in severe COVID patients with acute respiratory distress (143). As described here, there is substantial preclinical and clinical evidence that neuromodulation strategies may have a salutary impact on the host responses to the virus, and clinical trials to assess these strategies are warranted. Given the unabated SARS-CoV-2 pandemic, speed to execute these trials and deliver rigorous results is of essence.

The authors of this paper have formed the International Consortium on Neuromodulation for COVID-19 (ICNC; www.covidneuromod.org) with the mission to provide global leadership for the rapid advancement and clinical adoption of neuromodulation technologies to treat emerging infectious diseases. The ICNC envisions a global infrastructure capable of the rapid deployment and clinical validation of neuromodulation technologies for pandemics, epidemics, and other emerging public health crises for which they may be efficacious. Further, beyond applications for immunological and respiratory control in response to infection, neuromodulation has multiple potential therapeutic applications for the post-acute recovery phase. For example, a recent report has indicated that >87% of COVID-19 patients continue to experience at least one symptom following acute recovery (144), the most frequent of which was fatigue. Noninvasive brain stimulation approaches have reliable benefit for fatigue (145), as well as for psychological symptoms such as depression and anxiety that are common in the recovery period (146–148).

ICNC members have developed recommended endpoints for neuromodulation clinical in COVID-19, as well as recommended publishing guidelines to describe the technology tested.

While prospective, RCTs are the gold standard for such clinical trials and sham control arms are desirable, they might not always be necessary if objective endpoints are used to measure outcomes (149). Such endpoints will vary based on hypothesized mechanism of action of the technology, as well as the patient population being studied: wearable and handheld neuromodulation technologies can be trialed in hospitalized patients (non-ICU or ICU), as well as ambulatory outpatients pre- or post-hospitalization. Nevertheless, some key data elements and outcome measures are recommended, in the anticipation that post-study meta-analyses can illuminate overarching trends not apparent in individual studies (Table 6). Upon publication of study results, the ICNC members recommend careful descriptions of the neuromodulation technology (150).

**Table 6:** Recommended clinical trial endpoints for studies of neuromodulation technologies in COVID-19.

**Baseline demographics/signs & symptoms**	**Age**
	Gender
	BMI
	Comorbidities
	Tobacco/vape use
	Date of known or suspected exposure to virus
	Date of first positive diagnostic test
	Days since symptom onset/discharge
	Duration of symptoms
	Cough, on a patient-reported scale of severe, moderate, mild, absent
	Oxygen saturation level (SpO2)
Inpatient measures	Emergency severity index at presentation
	Blood pressure (systolic/diastolic)
	Time on supplemental oxygen
	Spirometry (Liu et al.)
	Incidence of non-invasive positive pressure ventilation or heated high flow nasal canula use
	Days on non-invasive positive pressure ventilation or heated high flow nasal canula use
	Occurrence of ICU transfer from non-ICU hospital bed and time to transfer
	Duration of ICU stay in days
	Occurrence of mechanical ventilation
	Days to and on mechanical ventilation
	Time to clinical recovery defined as the time (in hours) from initiation of therapy until normalization of fever, respiratory rate, and oxygen saturation, and alleviation of cough, sustained for at least 72 hours. Normalization and alleviation criteria as follow:
	Fever - ≤ 36.6°C or -axilla, ≤ 37.2°C oral or ≤ 37.8°C rectal or tympanic
	Respiratory rate - ≤ 24/min on room air
	Oxygen saturation - >94% on room air
	Cough - mild or absent on a patient-reported scale of severe, moderate, mild, absent
	Hospital length of stay (LOS)
	All-cause mortality
Outpatient measures	Body temperature, twice daily (morning and evening)
	Ambulatory spirometry or breathing measures
	Headache/pain scores and BAS
	Respiratory rate
Mechanistic measures	Inflammation: TNF, IL-1b, IL-6, IL-8, IL-10, IFNg, CRP. Ferritin
	Pulmonary function: forced expiratory volume

*Study designers are encouraged to ensure specific biomarkers to the technology and hypothesized mechanism of action are included*.

We strongly believe that multi-national and academic-industry collaborative and coordinated efforts to tackle COVID-19 are necessary to advance our knowledge and provide the urgently needed therapeutic options. Our present collaboration and this review on neuromodulation will hopefully serve as an example/roadmap for other scientific areas.

## Author Contributions

All authors wrote sections of the manuscript, contributed to manuscript revision, and read and approved the submitted version.

## Conflict of Interest

CC is an equity holder of Convergent Medical Technologies, Inc., reports personal fees from electroCore Inc., and Spark Biomedical, and has issued and pending patents related to vagus nerve stimulation to control bleeding and inflammation. MB reports personal fees from Soterix Medical, grants from Google X, personal fees from Halo Neuroscience, outside the submitted work; in addition, he has a patent Brain Stimulation issued. MFr reports pending patent applications describing injectable electrode structures for Neuronoff, Inc., and is an employee and equity holder of Neuronoff, Inc. He further reports issued patents for selective Vagus nerve stimulation and selective electrical vagal block for regulation of autonomic functions such as heart rate and blood pressure for Boston Scientific. MFu reports personal fees from Axon Therapies, CVRx, Daxor, Edwards Life Sciences, Galvani, and Respicardia, outside the submitted work. SH is an employee and equity holder of Cala Health. NK reports a patent devices and methods for reducing inflammation using electrical stimulation pending. BS reports personal fees from electroCore, and reports patents issued and pending related to transcutaneous cervical vagus nerve stimulation. PS is an employee and equity holder of electroCore, and reports patents issued and pending related to transcutaneous cervical vagus nerve stimulation. The remaining authors declare that the research was conducted in the absence of any commercial or financial relationships that could be construed as a potential conflict of interest.

## Publisher's Note

All claims expressed in this article are solely those of the authors and do not necessarily represent those of their affiliated organizations, or those of the publisher, the editors and the reviewers. Any product that may be evaluated in this article, or claim that may be made by its manufacturer, is not guaranteed or endorsed by the publisher.
